# Wild Blackberry Fruit (*Rubus fruticosus* L.) as Potential Functional Ingredient in Food: Ultrasound-Assisted Extraction Optimization, Ripening Period Evaluation, Application in Muffin, and Consumer Acceptance

**DOI:** 10.3390/foods13050666

**Published:** 2024-02-22

**Authors:** Beatrix Sik, Zsolt Ajtony, Erika Lakatos, Rita Székelyhidi

**Affiliations:** Department of Food Science, Albert Kázmér Faculty of Agricultural and Food Sciences of Széchenyi István University, 15-17 Lucsony Street, 9200 Mosonmagyaróvár, Hungary; ajtony.zsolt@sze.hu (Z.A.); lakatos.erika@sze.hu (E.L.); szekelyhidi.rita@sze.hu (R.S.)

**Keywords:** ultrasound-assisted extraction, blackberry fruit, antioxidant compounds, functional food

## Abstract

The aim of the present study is to evaluate the antioxidant properties of wild blackberry fruits as well as their possible use in powdered form as a functional ingredient. For this, ultrasound-assisted extraction optimization, ripening stage evaluation, and wild blackberry powder incorporation into a real food matrix were applied. The optimum conditions for extraction were as follows: 60% MeOH, 20 min of extraction time, acidification with 0.5% HCl, and a 1:40 g/mL solid-to-solvent ratio, which allowed the following yields: total polyphenol content (TPC): 53.8 mg GAE/g; total flavonoid content (TFC): 5.78 mg QE/g; total monomer anthocyanin content (TMA): 11.2 mg CGE/g; 2,2-diphenyl-1-picrylhydrazyl radical scavenging activity (DPPH): 71.5 mg AAE/g; IC_50_: 52.3 µg/mL. The study also highlighted that, during the ripening process, the TPC (41.4%), TFC (17.0%), and DPPH levels (66.4%) of the fruits decreased while the TMA yield increased. The incorporation of blackberry powder at different levels (5–20%) increased the TPC, TFC, TMA, and antioxidant properties of muffins. Although the muffins enriched with 20% wild blackberry powder had the best chemical properties (TPC: 3.15 mg GAE/g; TFC: 0.52 mg QE/g; TMA: 0.23 mg CGE/g; DPPH: 1.70 mg AAE/g; IC_50_: 1.65 mg/mL), the sensory analysis showed that the addition of blackberry fruit at a concentration of 10% to the muffins resulted in the best consumer acceptability.

## 1. Introduction

Although wild fruits are often underutilized and less known, in recent decades, several studies have shown that they can be a potential source of bioactive phytochemicals, including phenolic acids, anthocyanins, and vitamins [1,2,3]. In general, wild fruits are smaller, contain more seeds, and, in terms of shape, they are not as round as their cultivated versions. The main advantage of wild fruits is that they are more resistant to extreme weather conditions and are immune to many diseases [4]. Wild blackberry (*Rubus fruticosus* L.) is a perennial and thorny subshrub that belongs to the Rosaceae family. It grows naturally beside forests, ditches, and uncultivated fields. Although wild blackberry is native to Europe and Asia, it can be commonly found in temperate climates. Its pink-white flowers bloom in May, followed by its blackish-blue fruit [5]. Wild blackberry has been used in folk medicine for the treatment of cough, toothache, and diarrhea [6]. These beneficial effects are connected to phenolic compounds such as cyanidin-3-glucoside, cyanidin-3-galactoside, quercetin-3-galactoside, quercetin-3-glucoside, *p*-hydroxybenzoic acid, and gallic acid [7]. Wild blackberry fruits are often consumed freshly but can be used for the production of different types of foods, such as ice cream, jam, cake, or juice [8]. The term functional food is used to describe healthy foods or food components that offer benefits beyond adequate nutritional effects and may improve health [9]. Bioactive compounds are usually responsible for the functionality of such foods. In the plant kingdom, they are referred to as phytochemicals, i.e., chemical compounds produced by plants. Currently, the use of secondary plant metabolites in foods has received increasing attention because of their beneficial effects on the human body [10]. Earlier studies [11,12] highlighted that the consumption of phenolic-rich foods may play a critical role in the prevention of several chronic diseases due to their antioxidant properties. Although berry fruits are a rich source of antioxidant compounds, they are highly perishable. Therefore, it is essential to pay more attention to the retention of health-protective antioxidant compounds from fruits at post-harvest stages [13].

Muffins are one of the most popular bakery products due to their ready-to-eat nature and affordable cost. Among the baked breakfast products, muffins rank third. They are available in different varieties, thereby attracting a broad range of consumers [14]. However, due to their ingredients (wheat flour, sugar, fat, eggs), muffins are considered high-calorie sweet products. Currently, people are becoming more conscious of health and nutrition, so it is important to enhance these types of products with valuable ingredients to make them healthier [15]. In recent years, several studies have proven that blackberries can be an excellent functional food ingredient due to their bioactive compounds. For instance, dos Santos et al. [16] highlighted that blackberry pomace microcapsules are feasible for the food industry to produce functional dairy products. Overall, the developed yogurt enriched with blackberry contained a higher number of bioactive compounds, as well as had better bioavailability properties. Pereira et al. [17] confirmed that the fiber (830%), anthocyanin (2778%), and phenolic content (80%) of cookies can be significantly increased with blackberries.

Extraction is a key step for the recovery of phytochemical compounds. One of the most applied non-conventional techniques is ultrasound-assisted extraction (UAE) because it has many advantages, including less time and energy requirements and lower phytochemical compound degradation [18]. The main mechanism of UAE is acoustic cavitation. During the extraction, the ultrasonic waves cause the formation of cavitation bubbles that may enlarge and collapse, which results in fragmentation, pore formation, and shearing in the cellular matrix of the plant, along with other complex phenomena. This simplifies the release of phenolic compounds, resulting in an increased extraction efficiency. However, there are many parameters (temperature, solvent, extraction time, solid-liquid ratio, etc.) that may affect the maximum recovery of the bioactive compounds during extraction [19]. Therefore, this study aimed to maximize the yield of antioxidant compounds from wild-grown blackberry fruits by UAE. Another aim was to assess the effect of blackberry powder on the antioxidant and consumer acceptance properties of muffins.

## 2. Materials and Methods

### 2.1. Chemicals

The Folin–Ciocalteu reagent, gallic acid, sodium acetate, absolute ethanol, potassium chloride, formic acid, 2,2-diphenyl-1-picrylhydrazyl (DPPH), aluminum chloride, quercetin hydrate, anhydrous sodium carbonate, and L-ascorbic acid were purchased from Merck Life Science Ltd. (Budapest, Hungary). Methanol was provided by Reanal Laboratory Chemicals Ltd. (Budapest, Hungary), while hydrochloric acid was supplied by Biolab Inc. (Budapest, Hungary). High-purity deionized water (18 MΩ cm) was generated by a Zeener Power I (Human Corporation, Seoul, Korea) system.

### 2.2. Plant Material

Wild-growing blackberry fruits (*Rubus praecox* Bertol.) were harvested in Hegyeshalom, Győr-Moson-Sopron County, Hungary (coordinates: 47°54′54.07″ N, 17°9′17.6″ E) in August 2022. The identification was based on the currently available taxonomic studies [20,21]. Three ripening periods were examined as follows: unripe (green color), semi-ripe (red color), and ripe (blackish-blue color). It is well-known that the convective drying method does not require large financial outlays or complicated equipment. In addition, this process is characterized by low energy efficiency. Therefore, the fruits were dried to constant weight in a food dehydrator (FDK24DW, Gorenje, Velenje, Slovenia) at 50 °C for 10 h. Afterward, the samples were ground with a coffee grinder (CFFG-200, Dyras, Veszprém, Hungary) to a fine powder and stored in a vacuum-sealed bag at −18 °C until chemical analysis. The ground powder was used as flour for muffin preparation.

### 2.3. Optimization Ultrasound-Assisted Extraction (UAE) Procedure

**Figure 1:**
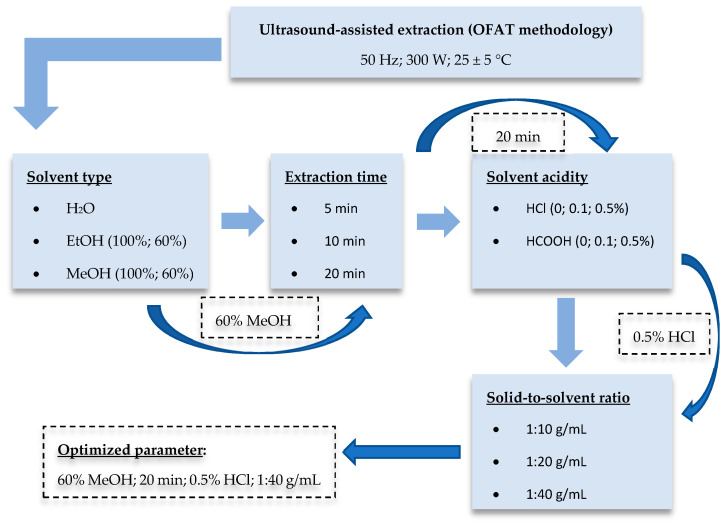
Schematic diagram of the optimization procedure.

The optimization step (Figure 1) was planned using the one-factor-at-a-time (OFAT) methodology. In initial screening experiments, EtOH-H_2_O (0:100, 60:40, 100:0, *v*/*v*) and MeOH-H_2_O (0:100, 60:40, 100:0, *v*/*v*) mixtures were tested to investigate the effect of solvent type on the yield of extractable antioxidant compounds. To choose the appropriate extraction solvent, the extraction time was set for 20 min, and the solid-to-solvent ratio was 1:40 g/mL. For further optimization steps, three different extraction times (5, 10, and 20 min) were tested. Taking into account the results, 60:40 *v*/*v* MeOH-H_2_O mixture and the extraction time of 20 min were further used to determine the influence of acidification. For this, HCl and HCOOH were applied at three different concentration (0, 0.1, and 0.5%) levels. Finally, the impact of the solid-to-solvent ratio (1:10, 1:20, and 1:40 g/mL) was examined. Treatments were carried out at 25 ± 5 °C in all cases. The power of the ultrasonic bath (UC002BM1, Tesla, Prague, Czechoslovakia) was 300 W (frequency: 50 Hz). After the UAE procedure, the samples were centrifuged (Z206A, Hermle, Wehingen, Germany) at 6000 rpm for 15 min, and supernatants were diluted with high-purity deionized water for the spectrophotometric analysis.

### 2.4. Muffin Preparation

Using a modified version of the recipe published by Tukassar et al. [22], muffins were prepared using a combination of the following ingredients: sugar (22%), margarine (14.7%), flour (29.4%), milk (17.6%), eggs (15.6%), and baking powder (0.7%). Wild blackberry fruit powder was incorporated into muffins at 4 levels (0%, 5%, 10%, and 20%) by replacing an equivalent amount of flour. Sugar was mixed with margarine for around 5 min with an electric hand mixer (SHM300C1, SilverCrest, Bochum, Germany). Then, whisked eggs were added to the sugar–margarine mixture. Flour, baking powder, and blackberry fruit powder were sifted and added slowly to the sugar–margarine–eggs mixture. Finally, the milk was poured into the mixture and stirred continuously for 2 min. A total of 40 ± 1 g batter was weighed in muffin papers and baked (W7OM44S1PBL, Whirlpool, Benton Harbor, MI, USA) in a muffin pan at 180 °C for 25 min.

### 2.5. Extraction of Muffin Samples

The sample preparation procedure consisted of two different steps. First, all samples were dried in a food dehydrator (FDK24DW, Gorenje, Velenje, Slovenia) at 50 °C for 6 h. Afterward, the dried samples were pulverized to a fine powder in a mortar and pestle and homogenized by a kitchen stainless sieve. It is well known that phenolic compounds are well soluble in high- or medium-polarity organic solvents. In addition, the interactions between polyphenols and food macronutrients can cause a multitude of problems in the analysis, including the generation of emulsions or sample turbidity. Therefore, in the second step, the homogenized samples (2 g) were defatted with n-hexane (20 mL). For this, the samples were vortexed for 1 min in a Falcon tube, then sonicated for 5 min in an ultrasonic bath at room temperature (frequency: 50 Hz, power: 300 W). Afterward, the mixtures were centrifuged for 15 min at 6000 rpm, and the supernatants were removed. Finally, the defatted muffins were dried at ambient temperature for a night and used for the extraction. For the extraction, the best conditions found in the optimization step were used, except for the sample weight/solvent ratio. All in all, 15 mL of 60:40:0.5 *v*/*v*/*v* MeOH-H_2_O-HCl mixture was added to a 1.5 g sample, which was then sonicated (frequency: 50 Hz, power: 300 W) for 20 min. After the UAE procedure, samples were centrifuged as mentioned above, and the supernatants were used without dilution for spectrophotometric analysis.

### 2.6. Phytochemical Analysis

Measurements of the extracts and muffin samples were performed with a Spectroquant Pharo 100 (Merck, Darmstadt, Germany) spectrophotometer. To measure total polyphenol content (TPC), the Folin–Ciocalteu reaction [23] was used with gallic acid as the standard, and results were evaluated (wavelength: 725 nm) in terms of gallic acid equivalents (mg GAE/g). Briefly, a 50 µL sample was mixed with 1.5 mL of water, 2.5 mL of Folin-reagent (10 *v*/*v*%), and 2 mL of sodium carbonate solution (7.5 g/100 mL). The samples were allowed to stand in the dark at room temperature for 90 min. TMA content was examined using the pH differential method [24], and results were expressed as cyanidin 3-glucoside equivalents (mg CGE/g). In brief, 0.5 mL of the sample was mixed with 4.5 mL of potassium chloride (25 mM, pH = 1.0) and sodium–acetate buffer (0.4 M, pH = 4.5), respectively. Each mixture reacted at room temperature for 15 min. The absorbance was read at 520 and 700 nm. DPPH radical scavenging activity assay [25] was carried out using 0.1 mM of DPPH solution (3 mL), and results were expressed as ascorbic acid equivalents (mg AAE/g). For the analysis, a 100 µL aliquot of sample was reacted with DPPH solution at room temperature for 30 min. The samples were analyzed at 517 nm. In addition, the half-inhibitory concentration (IC_50_) was obtained through the interpolation of linear regression analysis. The TFC was assessed by using a method proposed by Assefa et al. [26], i.e., 0.5 mL of extracts, 1.5 mL of ethanol, 100 µL of aluminum chloride (10%), 100 µL of sodium-acetate (1 M), and 2.8 mL of high-purity water were reacted at room temperature for 30 min. Absorbance was measured at 415 nm, and data were expressed as quercetin equivalents (mg QE/g).

### 2.7. Consumer Acceptance

The sensory acceptability evaluation was performed on freshly prepared muffin samples. A muffin without blackberry powder was used for sensory comparison. The panelists consisted of 21 untrained tasters (ages ranging from 19 to 58) of the University community (Department of Food Science at Széchenyi István University). The tasters were selected based on interest, time availability, and the absence of allergies to the muffin ingredients used during baking. The sensory attributes were evaluated by using a five-point hedonic scale, where 1 corresponded to dislike very much, 3 to neither like nor dislike, and 5 to like very much. Tasters were asked to rinse their mouths with tap water between each muffin sample. The sensory characteristics of muffins were evaluated according to the study reported by Rodríguez et al. [27] with slight modifications. The descriptors evaluated were appearance (external and internal color, muffin dome shape, air bubbles in the crumb), texture (hardness, elasticity, adhesiveness), odor (typical muffin odor, fruit odor), taste (sweetness, typical muffin flavor, fruit flavor, off-flavor), and overall acceptability (according to the preference).

### 2.8. Statistical Analysis

One-way analysis of variance (ANOVA) followed by Tukey’s multiple comparison test at *p* < 0.05 level was used to compare the significant difference in the data. All measurements were performed in triplicate. The final results were given as the mean ± standard deviation (SD). Data were analyzed using Microsoft Excel software (2016). The XLSTAT (2024) software was used for principal component (PCA) analysis and agglomerative hierarchical clustering (AHC).

## 3. Results and Discussion

### 3.1. Optimization of Ultrasound-Assisted Extraction (UAE)

#### 3.1.1. Effect of Solvent Type

**Figure 2:**
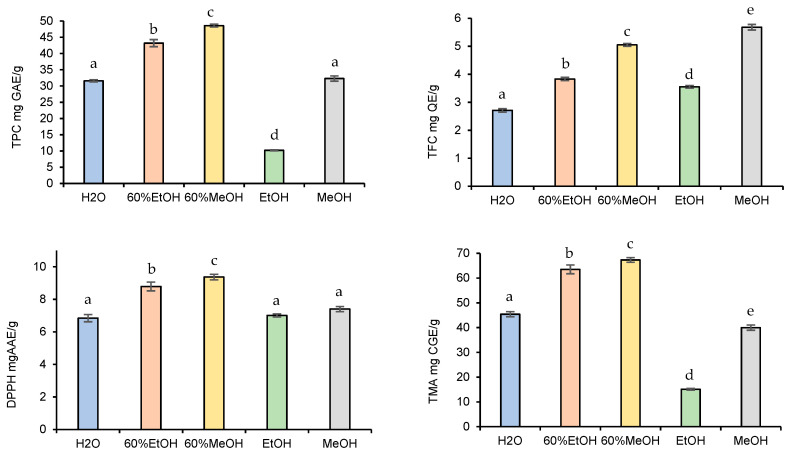
Effect of solvent type on TPC, TFC, TMA, and DPPH yield. Data presented as mean ± SD of measurements. Different letters indicate significant differences.

To recover the maximum possible quantity of phenolic compounds from fruits, the optimization of the extraction process is indispensable. Therefore, in this study, various independent factors that may affect the yield of extractable phenolic compounds were evaluated. It is well-known that the solvent type with varying polarities and the chemical nature of plant material highly affect the extractable phenolic concentration [28]. The effects of the solvent type on phenolic extraction were examined first (Figure 2).

When observing the effects of the solvent type on the TPC, TFC, TMA contents, and DPPH antioxidant activities of the blackberry fruits, 60% alcoholic mixtures exhibited higher extractable yield than the H_2_O or pure EtOH and MeOH. At the same time, the amount of the extractable phytochemical compounds was significantly higher in the 60% MeOH extract than in the 60% EtOH. Overall, the extracted TPC, TFC, TMA, and DPPH yields obtained by 60% MeOH were as follows: 48.6 ± 0.42 mg GAE/g, 5.05 ± 0.05 mg QE/g, 9.37 ± 0.17 mg CGE/g, and 67.3 ± 0.94 mg AAE/g. All in all, the obtained results were in agreement with the previously published results [19], i.e., mixtures of alcohols and water are more effective in extracting phenolic compounds compared to the pure solvent systems.

The determination of antioxidant radical scavenging activities by determination of the efficiency concentration inhibiting 50% of DPPH molecules also reinforced the fact that the 60% MeOH gave the best recoveries (56.1 µg/mL). The IC_50_ value was also low for 60% EtOH (57.8 µg/mL), followed by H_2_O (95.1 µg/mL), MeOH (102 µg/mL), and EtOH (265 µg/mL). Among the pure solvents, MeOH was the most efficient medium for the extraction of antioxidant ingredients. Overall, the best yields were obtained with 60% methanol, except for TFC; therefore, it was used for further experiments. These findings were similar to those obtained (63.7% MeOH) by Espada-Bellido et al. [29], who optimized the UAE using response surface methodology.

#### 3.1.2. Effect of Sonication Time and Acidification

Choosing an appropriate extraction time was the second step in the optimization study (Table 1). The main advantage of using ultrasound is that it allows for a more efficient extraction of bioactive compounds in a shorter time than conventional methods such as maceration [30]. Sonication was applied for 5, 10, and 20 min. Results clearly show that as the extraction time was increased, the TPC, TFC, and the antioxidant properties of the blackberry extracts were also increased. The highest change (20%) was detected in the yield of TPC when the extraction time was increased from 5 min to 20 min. A similar trend was seen for TFC (13.2%) and DPPH radical scavenging activity measurements (15.6%). Similar to us, Ivanovic et al. [31] observed that increased sonication time (from 15 to 30 min) has a positive effect on the extraction efficiency of extractable antioxidant compounds from blackberry fruits. However, as can be seen from Table 1, in the case of TMA, the longer extraction time caused degradation in the yield. This effect is assumed to be caused by cavitation occurring during the sonication process. In addition, anthocyanins may react with free radicals produced during sonication, thus helping the breakdown of the structure of the pigments [32].

To improve the extraction efficiency, the solvent can be acidified. For the best extraction efficiency of phenolic compounds, it is recommended to use weak organic acids (e.g., formic acid or acetic acid) or low concentrations of strong acids (e.g., HCl) [32,33]. The effect of acidification on extractable antioxidant compounds was examined after 20 min of sonication time. It is well known that acidification often plays an important role in phenolic compound extraction, especially for anthocyanins. Similar to the study by Stanoeva et al. [34], we found that HCl gave a significantly higher yield than formic acid, which can be explained by the higher amount of anthocyanin compounds since it is well established that these compounds occur in different chemical form in media with different pHs.

As shown in Table 1, the variable HCOOH concentration did not significantly affect the TMA and DPPH. At the same time, a significant increase was observed for TPC and TFC at higher concentrations (0.5%). In the case of HCl, a concentration of 0.5% provided a significant increase in phytochemical recoveries. The highest change was observed for TMA, with an increase of 19.5%, followed by TFC (14.5%), TPC (10.7%), and DPPH (6.2%).

#### 3.1.3. Effect of Solid-to-Solvent Ratio

The final step was to evaluate the effect of the solid-to-solvent ratio on the extraction efficiency (Table 2) since it is well-known that this parameter can highly affect the extractable yield. In this study, three different solid-to-solvent ratios (1:10, 1:20, and 1:40 g/mL) were investigated while the extraction solvent (60% MeOH containing 0.5% HCl) and time (20 min) were kept constant.

The highest recoveries of TPC, TFA, TMA, and DPPH were observed at 1:40 g/mL with the following yields: 53.6 ± 0.59 mg GAE/g, 5.78 ± 0.08 mg QE/g, 11.2 ± 0.41 mg CGE/g, and 71.5 ± 0.96 mg AAE/g, respectively. As shown in Table 2, the lowest yields were obtained at a 1:10 g/mL sample solid-to-solvent ratio. These results suggested that a higher solid-to-solvent ratio increases the concentration gradient between the plant material and the solvent phase, which enhances the dissolution and diffusivity of phenolics in the solvent [35].

### 3.2. Ripening Period Evaluation

**Figure 3:**
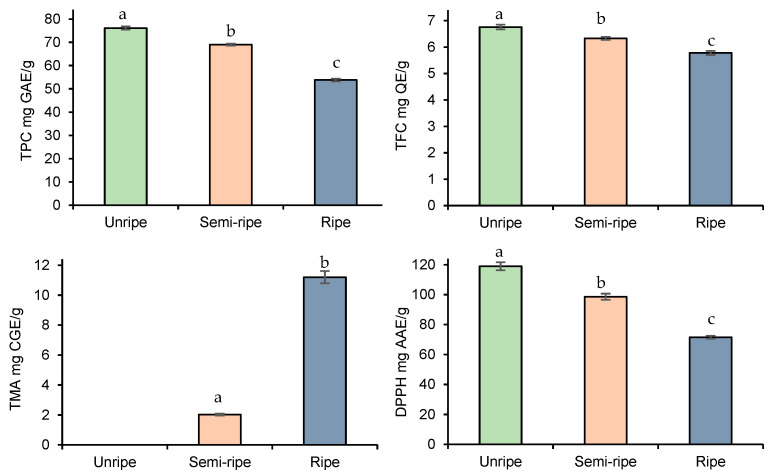
Effect of ripening stage on TPC, TFC, TMA, and DPPH yield. Data presented as mean ± SD of measurements. Different letters indicate significant differences.

After the optimization of extraction parameters, we further deepened our study, investigating how the ripening process may affect the bioactive compound accumulation in blackberry fruits. As indicated in Figure 3, TPC, TFC, and DPPH decreased, while the TMA increased with the progress of fruit ripening.

In this regard, the observations were in agreement with those found in previous studies [36,37,38], i.e., anthocyanins accumulate in the highest concentration at the end of the ripening process while the other antioxidant compounds decrease. The high concentration of phenolic compounds in unripened fruits can be explained by fruit protection from various fruit-borne diseases during pre-maturation. On the other hand, as the fruits proceed toward maturation, their phenolic compounds may contribute to the biosynthesis of the flavylium ring of anthocyanins [39]. All in all, the highest decrease was observed for DPPH scavenging activities of fruits (from 119 ± 2.66 mg AAE/g to 71.5 ± 0.96 mg AAE/g), followed by TPC (from 76.1 ± 0.69 mg AAE/g to 53.8 ± 0.59 mg AAE/g) and TFC (from 6.76 ± 0.09 mg AAE/g to 5.78 ± 0.08 mg AAE/g).

Pearson’s correlation coefficient (r) was also conducted to express the relationship between the TPC, TFC, TMA, and DPPH radical scavenging activities. A strong positive correlation was found between TPC, TFC, and antioxidant activities with correlation coefficients of r = 0.992 and r = 0.990, respectively, while negative linear correlations were detected between TMA and antioxidant activities (r = −0.963).

### 3.3. Fortified Muffin Characterization

Previously, Jazić et al. [40] reported that the pomaces from wild blackberries had higher-yield phenolic compounds and stronger biological potential and, thus, could be a promising food additive, natural antioxidant, and colorant in the food industry. The TPC, TFC, and TMA yields and the DPPH radical scavenging activities of the fortified muffins are presented in Table 3, which squarely supports that the blackberry powder fortification was able to significantly (*p* < 0.05) increase the contents of bioactive compounds in muffins. These findings were also confirmed by other types of food, including yogurt [16], cookies [17], kefir [41], and chocolate [42].

While the TPC was 0.20 mg GAE/g in the control muffin, the substitution of wheat flour for blackberry powder resulted in a significant increase (167%) in TPC values from 1.18 mg GAE/g to 3.15 mg GAE/g with the increase of blackberry powder levels from 5% to 20%. The same effect was found for TFC yields. In this case, the TFC increased from 0.20 mg QE/g to 0.52 mg QE/g, when the concentration of blackberry powder was increased from 5% to 20%. Moreover, a significant increase was recorded for TFC (160%), TMA (53.3%), and DPPH radical scavenging activity (25.0%) yields when the concentration of blackberry powder was increased from 5 to 20%. It is also important to note that the yields of bioactive compounds did not increase proportionately with the increasing amount of added powder. This phenomenon may be associated with the other ingredients in muffins, such as proteins, lipids, or sugars, which can react with phenolic compounds [43]. In addition, food processing may also affect the varied behaviors of anti-oxidant ingredients [44].

The Pearson correlation coefficient between the investigated parameters was high for muffin samples. All in all, the r-values between TPC, TFC, TMA, and the antioxidant activities were as follows: 0.936, 0.954, and 0.863, respectively.

### 3.4. Acceptance Evaluation

**Figure 4:**
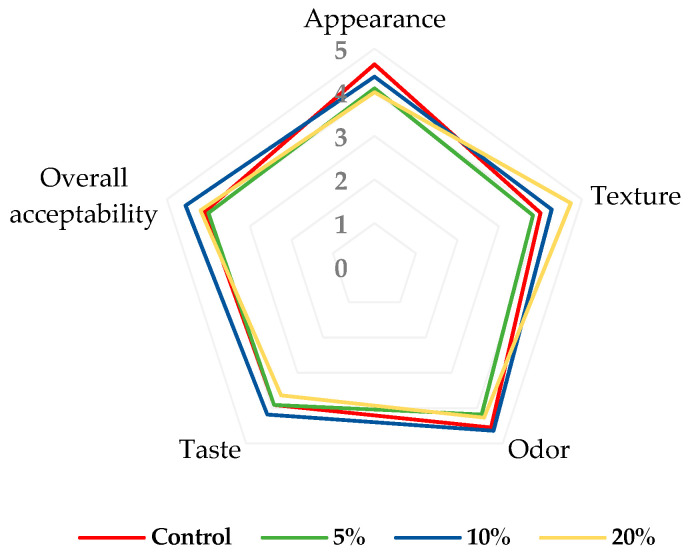
Consumer acceptance evaluation of muffins; 5%—enriched with 5% blackberry powder; 10%—enriched with 10% blackberry powder; 20%—enriched 20% blackberry powder.

The acceptance evaluation of the control and fortified muffins can be seen in Figure 4.

**Figure 5:**
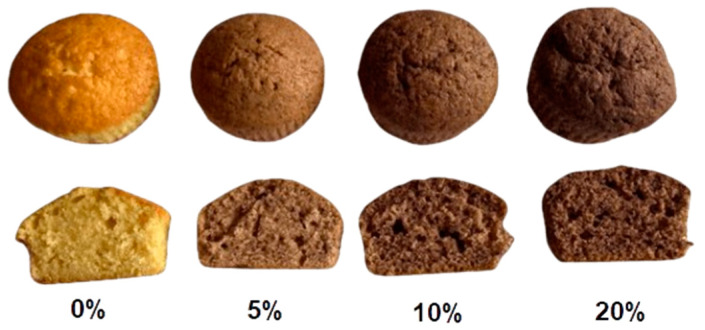
Muffin samples with different concentrations of blackberry powder.

Overall, among the fortified muffins, the sample with 10% blackberry powder showed the highest acceptability scores for evaluated attributes, particularly in appearance (4.36), odor (4.64), taste (4.18), and overall acceptability (4.55). In addition, muffins with 20% blackberry powder recorded the highest texture score (4.73). In this case, some tasters stated that the texture of the muffins was much softer. At the same time, these samples (muffins with 20% blackberry powder) had the lowest scores for appearance (4.00) and taste (3.64). All in all, the results showed that all fortified muffin samples had satisfactory scores, between 3.64 and 4.55 on a 5-point scale (Figure 5). Only the muffins with 5% blackberry powder seem to have lower acceptability, especially due to their texture and taste.

### 3.5. Principal Component Analysis (PCA) and Agglomerative Hierarchical Clustering (AHC)

**Figure 6:**
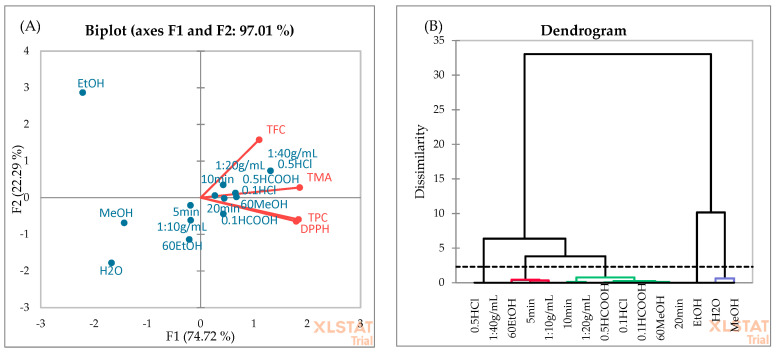
PCA (**A**) and AHC (**B**) analyses of independent factors used for the OFAT optimization.

PCA (Figure 6A) and AHC (Figure 6B) analyses were employed to evaluate the association among the independent variables used for OFAT. According to PCA, the first two principal components explain 97.01% of the total variance, which confirms that these principal components are adequate to describe the variation among all data. The first and second components account for 74.72% and 22.29% of the variability, respectively. The dendrogram showed five distinct classes. H_2_O and MeOH solvent types form the first separated class, while in the second class, three different independent factors (solvent type: 60%EtOH, extraction time: 5 min, sample-to-solvent ratio: 1:10 g/mL) were placed. In the third cluster, 0.5% HCOOH and 0.1% HCl showed the highest similarity. This is consistent with the data in Table 3, as no significant difference was found between the extraction efficiency of these acids. Pure EtOH formed the fourth cluster, which may be due to lower TPC and TMA yields in blackberry fruits (Figure 2). Cluster 5 included the 0.5% HCl and 1:40 g/mL sample-to-solvent ratio. The best extraction yields were observed when the extraction solvent (60%MeOH) was acidified with 0.5% HCl using a 1:40 g/mL sample-to-solvent ratio (Table 2).

**Figure 7:**
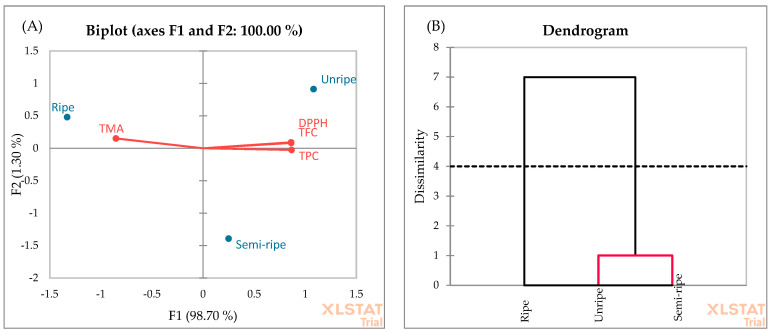
PCA (**A**) and AHC (**B**) analyses of ripening stages.

PCA was also applied to evaluate the data of antioxidant properties determined by TPC, TFC, TMA, and DPPH radical scavenging activity of blackberry fruit in different ripening stages (Figure 7A). The F1 and F2 explain 98.70% and 1.30% of the total variance, respectively. Moreover, the cumulative percentage of the two first principal components is 100%. In the F1 factor space, the unripe and semi-ripe fruits correlated positively with TPC, TFC, and DPPH radical scavenging activity, while the ripe fruits correlated positively with TMA. The negative zone of F1 is integrated by the TMA. As shown in Figure 7A, the presence of ripe fruit can be observed near this score. These observations highlight that the ripe fruits exhibited the highest TMA yield compared to the unripe and semi-ripe fruits. This finding is consistent with the results obtained in Figure 3. All in all, the F1 axis is principally constructed by the positive correlation between the TPC, TFC, and DPPH and the negative correlation between TMA. The AHC analysis (Figure 7B) showed two groups. Unripe and semi-ripe fruits formed the first cluster. This similarity may be due to higher TPC, TFC, and DPPH yields at the beginning of the ripening stage.

**Figure 8:**
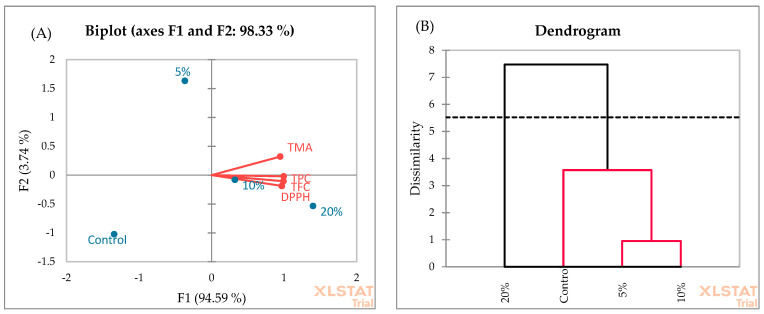
PCA (**A**) and AHC (**B**) analyses of muffin samples.

The PCA analysis of muffins (Figure 8A) showed that F1 and F2 explain 97.33% of the total variance. F1 explains 94.59% of the variance compared to 3.74% for the second axis (F2). The F1 axis is principally constructed by the positive correlation between all investigated chemical parameters. The similarity of muffin samples was evaluated using AHC, and two clusters were suggested (Figure 8B). Cluster 2 was characterized by muffins enriched with 20% blackberry fruit. This cluster showed the highest yields of TPC, TFC, TMA, and DPPH radical scavenging activity. In cluster 1, muffins enriched with 5% and 10% blackberry fruit showed the highest similarity, followed by control samples.

## 4. Conclusions

In the present study, a UAE method was successfully applied to extract antioxidant compounds from wild blackberry fruits. Four independent variables (solvent type, extraction time, solvent acidity, and solid-to-solvent ratio) were analyzed to determine the best extraction conditions. The best conditions obtained by OFAT optimization methodology are as follows: 60% MeOH, sonication time of 20 min, acidification with 0.5% HCl, and solid-to-solvent ratio of 1:40 g/mL. We also investigated the change of phytochemical compounds in blackberry fruit during three ripening stages. Overall, during the ripening process, the TPC, TFC, and DPPH levels of the fruits decreased while the TMA yield increased. Finally, blackberry powder was further used at different concentrations (0–20%) to develop functional muffins. All in all, we demonstrated that the fortification of muffins with blackberry powder may offer an opportunity to develop value-added muffins with higher antioxidant activity, TPC, TFC, and TMA. Therefore, these results suggest that wild blackberry fruits may play an important role in environmental sustainability as they grow wildly and can be used as a functional ingredient. The best consumer acceptance was observed when the muffins were enriched with 10% wild blackberry powder. However, this study has some weaknesses that must be acknowledged. First, the chosen optimization methodology does not address interactions among independent factors (e.g., solvent type, extraction time). Second, spectrophotometric methods cannot identify the individual bioactive compounds. Therefore, more attention is currently being paid to the chromatographic analysis of phenolic compounds from plants.

## Figures and Tables

**Table 1:** Effect of extraction time and acidification on TPC, TFC, TMA, and DPPH yield.

	TPC (mg GAE/g)	TFC (mg QE/g)	TMA (mg CGE/g)	Antioxidant Activity	
				DPPH (mg AAE/g)	IC_50_ (µg/mL)
Extraction time (min)					
5	40.5 ± 0.70 ^a^	4.46 ± 0.02 ^a^	9.06 ± 0.14 ^a^	58.2 ± 0.81 ^a^	67.2 ± 0.65 ^a^
10	45.5 ± 0.74 ^b^	4.82 ± 0.07 ^b^	9.79 ± 0.28 ^b^	60.5 ± 1.18 ^a^	63.3 ± 0.51 ^b^
20	48.6 ± 0.42 ^c^	5.05 ± 0.05 ^c^	9.37 ± 0.17 ^c^	67.3 ± 0.94 ^b^	56.1 ± 0.91 ^c^
Acidification (*v*/*v*%)					
0	48.6 ± 0.42 ^c^	5.05 ± 0.05 ^c^	9.37 ± 0.17 ^c^	67.3 ± 0.94 ^b^	56.1 ± 0.91 ^c^
HCOOH					
0.1	49.8 ± 0.49 ^d^	4.72 ± 0.09 ^d^	9.36 ± 0.14 ^c^	68.5 ± 1.45 ^b^	55.4 ± 0.49 ^c^
0.5	51.2 ± 0.80 ^d^	5.31 ± 0.08 ^e^	9.47 ± 0.21 ^c^	69.9 ± 1.1 ^b^	54.1 ± 0.53 ^c^
HCl					
0.1	50.6 ± 0.45 ^cd^	5.15 ± 0.12 ^ce^	9.77 ± 0.20 ^c^	69.6± 0.73 ^b^	54.7 ± 0.62 ^c^
0.5	53.8 ± 0.59 ^e^	5.78 ± 0.08 ^f^	11.2 ± 0.41 ^d^	71.5 ± 0.96 ^bc^	52.3 ± 0.59 ^d^

Data presented as mean ± SD of measurements. Different letters indicate significant differences in a column.

**Table 2:** Effect of solid-to-solvent ratio on TPC, TFC, TMA, and DPPH yield.

Solid-to-Solvent Ratio (g/mL)	TPC (mg GAE/g)	TFC (mg QE/g)	TMA (mg CGE/g)	Antioxidant Activity	
				DPPH (mg AAE/g)	IC_50_ (µg/mL)
1:10	44.2 ± 0.84 ^a^	4.00 ± 0.08 ^a^	9.53 ± 0.12 ^a^	52.4 ± 0.60 ^a^	73.2 ± 1.12 ^a^
1:20	48.7 ± 0.58 ^b^	5.10 ± 0.09 ^b^	10.0 ± 0.20 ^a^	58.0 ± 1.20 ^a^	69.2 ± 0.77 ^a^
1:40	53.8 ± 0.59 ^c^	5.78 ± 0.08 ^c^	11.2 ± 0.41 ^b^	71.5 ± 0.96 ^b^	52.3 ± 0.63 ^b^

Data presented as mean ± SD of measurements. Different letters indicate significant differences in a column.

**Table 3:** Effect of blackberry powder incorporation into muffins at different concentrations on the TPC, TFC, TMA, and DPPH antioxidant activity.

Sample	TPC (mg GAE/g)	TFC (mg QE/g)	TMA (mg CGE/g)	Antioxidant Activity	
				DPPH (mg AAE/g)	IC_50_ (mg/mL)
Control	0.20 ± 0.006 ^a^	0.09 ± 0.001 ^a^	-	1.36 ± 0.006 ^a^	3.15 ± 0.029 ^a^
5%	1.18 ± 0.027 ^b^	0.20 ± 0.005 ^b^	0.15 ± 0.004 ^a^	1.42 ± 0.013 ^b^	2.90 ± 0.025 ^b^
10%	1.64 ± 0.033 ^c^	0.30 ± 0.004 ^c^	0.17 ± 0.005 ^b^	1.61 ± 0.017 ^c^	2.39 ± 0.013 ^c^
20%	3.15 ± 0.022 ^d^	0.52 ± 0.006 ^d^	0.23 ± 0.007 ^c^	1.70 ± 0.009 ^d^	1.65 ± 0.007 ^d^

Data presented as mean ± SD of measurements. Different letters indicate significant differences in a column. - means TMA was not detected in the sample.

## Data Availability

The original contributions presented in the study are included in the article, further inquiries can be directed to the corresponding author.
